# Diagnosis and Management of Upper Tract Urothelial Carcinoma: A Review

**DOI:** 10.3390/cancers17152467

**Published:** 2025-07-25

**Authors:** Domenique Escobar, Christopher Wang, Noah Suboc, Anishka D’Souza, Varsha Tulpule

**Affiliations:** 1 Catherine and Joseph Aresty Department of Urology, University of Southern California, Los Angeles, CA 90089, USA; 2 Norris Comprehensive Cancer Center, University of Southern California, Los Angeles, CA 90007, USA; christopher.wang2@med.usc.edu (C.W.); anishkad@med.usc.edu (A.D.); varsha.tulpule@med.usc.edu (V.T.); 3 University of Southern California, Los Angeles, CA 90007, USA; suboc@usc.edu

**Keywords:** upper tract urothelial carcinoma, chemotherapy, immunotherapy, nephroureterectomy

## Abstract

**Background/Objectives:** Upper tract urothelial carcinoma (UTUC) is a rare and biologically distinct subset of urothelial malignancies, comprising approximately 5–10% of urothelial cancers. UTUC presents unique diagnostic and therapeutic challenges, with both a higher likelihood of invasive disease at presentation and a less favorable prognosis compared to urothelial carcinoma of the bladder. Current treatment strategies for UTUC are largely derived from bladder cancer studies, underscoring the need for UTUC-directed research. This review provides a comprehensive overview of UTUC, encompassing diagnostic approaches, systemic and intraluminal therapies, surgical management, and future directions. **Methods:** A narrative review was conducted synthesizing evidence from guideline-based recommendations, retrospective and prospective clinical studies, and ongoing trials focused on UTUC. **Results:** Neoadjuvant cisplatin-based chemotherapy is increasingly preferred in UTUC due to the risk of postoperative renal impairment that may preclude adjuvant cisplatin use. Surgical management includes kidney-sparing approaches and radical nephroureterectomy (RNU), with selection guided by tumor risk and patient comorbidities. While endoscopic management (EM) preserves renal function, it carries a higher recurrence and surveillance burden; RNU remains standard for high-risk cases. Systemic therapy for advanced and metastatic UTUC mirrors that of bladder urothelial carcinoma. Enfortumab vedotin (EV) plus pembrolizumab showed superior efficacy over chemotherapy in the EV-302 trial, with improved response rate, progression-free survival, and overall survival across subgroups, including UTUC. For patients ineligible for EV, the CheckMate-901 study supported first-line chemoimmunotherapy with gemcitabine, cisplatin, and nivolumab. Further systemic therapy strategies include maintenance avelumab post-chemotherapy (JAVELIN Bladder 100), targeted therapies such as erdafitinib (THOR trial), and trastuzumab deruxtecan (DESTINY-PanTumor02) in FGFR2/3-altered and HER2-positive disease, respectively. **Conclusions:** Historically, the therapeutic landscape of UTUC has been extrapolated from bladder cancer; however, ongoing research specific to UTUC is deriving more precise regimens involving the use of immune checkpoint inhibitors, antibody–drug conjugates, and biomarker-driven therapies.

## Simple Summary

Upper tract urothelial carcinoma is a rare cancer that forms in the lining of the kidney or ureter and is often diagnosed at a more advanced stage than bladder cancer. Because of its uncommon nature, diagnosing and treating this disease can be difficult, and most current clinical approaches still rely on research from bladder cancer studies. This review outlines modern diagnoses and management of upper tract urothelial carcinoma, including surgical and non-surgical options, as well as promising new therapies. Advances in diagnostic imaging and endoscopic tools have improved diagnosis, and treatment can range from kidney-sparing procedures to full removal of the kidney, ureter, and bladder cuff. Chemotherapy given before surgery may improve outcomes by addressing cancer early, while newer therapies like immunotherapy and gene-targeted treatments are under investigation for advanced cases. This review highlights a shift toward more personalized care, including efforts to integrate tumor biology and biomarkers to guide treatment decisions. These developments will hopefully improve outcomes for patients facing this uncommon but serious disease.

## 1. Introduction

Upper tract urothelial carcinoma (UTUC), urothelial cancer affecting the ureter, renal pelvis, and calyces, is a rare disease, accounting for only ~5–10% of all urothelial cancers [1]. Common presenting symptoms include hematuria or flank pain. Several risk factors have been identified, including male sex, smoking, alcohol use, nephrolithiasis, and exposure to certain toxins and chemicals [2,3]. Those with Lynch syndrome, a genetic syndrome caused by mutations in mismatch repair genes (MLH1, MSH2, MSH6, and PMS), also have an increased risk of developing UTUC compared to the general population, and these tumors tend to present in younger patients [4]. Those with UTUC also have a ~5% risk of contralateral disease [5] and a ~15–50% risk of developing urothelial carcinoma of the bladder (UCB) [2]. The risk of developing UTUC in those with bladder cancer ranges from 0.5 to 6% [6,7].

UTUC can be challenging to diagnose and manage, and those with UTUC more often present with invasive disease, and prognosis also tends to be poorer compared to those with UCB [3]. Much of the treatment for UTUC is extrapolated from UCB studies, and therefore, additional research is needed to improve outcomes. In this review, we provide a comprehensive overview of UTUC, including diagnosis, systemic, intraluminal, and surgical treatments, and future directions.

## 2. Diagnosis and Guideline Overview

The American Urological Association (AUA)/Society of Urologic Oncology (SUO) released guidelines for the management of non-metastatic UTUC in 2023 [8]. An important recommendation is that all patients with suspected UTUC should ideally undergo cross-sectional imaging with contrast and delayed images, cystoscopy, and ureteroscopy with biopsy and collection of urine cytology. Any concomitant bladder or urethral tumors should be managed in the same setting as the initial ureteroscopy. If ureteroscopy is not feasible, selective upper tract cytology may be obtained instead. If the contralateral upper tract is normal on preoperative clinical evaluation, it does not need to be evaluated intraoperatively. While diagnosis remains challenging, technological advances such as improvements in ureteroscopes and biopsy forceps have improved the ability to obtain an accurate diagnosis. At diagnosis, the patient should be risk-stratified according to the AUA risk groups (Table 1), which helps guide management and understand prognosis.

## 3. Neoadjuvant Therapy

Neoadjuvant cisplatin-based chemotherapy (NAC) is often delivered to cisplatin-eligible patients (ECOG 0-1, GFR > 50, hearing loss and peripheral neuropathy < grade 2, and New York Heart Association class < III heart failure), though there are no strict guidelines. The use of NAC in UTUC has been extrapolated from localized muscle-invasive bladder cancer (MIBC) data to target micro-metastatic disease with the goal of downstaging disease prior to radial nephroureterectomy (RNU). In the MIBC space, NAC prior to radical cystectomy (RC) showed a 5–8% improvement in OS at 5 years as compared to RC alone [9]. A 2021 meta-analysis of over 800 UTUC patients noted a pathological partial response rate of 43% and downstaging in 33% [10].

D’Andrea et al. retrospectively evaluated 1830 patients (1299 patients with UCB and 276 with UTUC) treated with NAC prior to RC or RNU. In total, 19.2% of the UCB patients had a pathologic complete response (pCR), defined as a post-treatment pathological stage ypT0N0, compared to 8.3% of the UTUC group (*p* < 0.01). A pathological objective response (pOR), defined as ypT0-Ta-Tis-T1N0, was seen in 40.3% of UCB patients and 48.2% of UTUC patients (*p* = 0.02). Amongst these patients who achieved pOR, 5-year overall survival (OS) and cancer-specific survival (CSS) were improved in the UTUC cohort. Furthermore, 5-year OS was 36% (95% CI 31–41) for UCB patients and 46% (95% CI 38–56) for UTUC patients, while CSS was 43% (95% CI 39–48) for UCB and 60% (95% CI 51–69) for UTUC patients [11].

Ideally, patients would receive NAC rather than adjuvant therapy, given the risk of impaired renal function with RNU. There is compelling evidence for the use of NAC to downstage UTUC patients prior to RNU. Coleman et al. conducted a multicenter, single-arm, phase II trial of high-risk UTUC treated with gemcitabine and split-dose cisplatin in the neoadjuvant setting. Of the 57 patients, 36 (63%) achieved a pathologic response, 11 (19%) of whom had a pCR (ypT0N0). Partial and complete responders attained improved progression-free survival (PFS) as compared to non-responders; 2-year PFS was 100% and 95% vs. 76% (*p* < 0.001), and there was an improved 2-year OS (100% and 100% vs. 80% (*p* < 0.001) [12].

A large, single-center, retrospective study evaluated 126 UTUC patients who received NAC: 62 received dose-dense methotrexate, vinblastine, Adriamycin, and cisplatin (ddMVAC); 28 received cisplatin with or without gemcitabine; 19 received gemcitabine, paclitaxel, and doxorubicin; and 17 with poor renal function received non-platinum-based regimens. The estimated median OS was 107 months (95% CI 86–125), while 5- and 10-year survival rates were 73.7% (95% CI 65.3–83.1) and 35.9% (95% CI 23.9–54). The 5- and 10-year CSS rates were 89.8% (95% CI 0.836–0.965) and 80.6% (95% CI 0.691–0.94). The 5- and 10-year metastasis-free survival (MFS) rates were 81% (95% CI 74–88.6) and 75.4% (95% CI 65.3–87). Results of this study emphasized long-term durable responses with the use of NAC prior to RNU in UTUC patients [13].

Margulis et al. conducted a multicenter ECOG-ACRIN-led prospective phase II study of NAC (either accelerated MVAC [aMVAC] or gemcitabine + carboplatin based on renal function). Of the 19 analyzed patients who received aMVAC prior to RNU, 14% (90% CI 4.9–28.8%) achieved a pCR, and 62% achieved ≤pT1. Importantly, post-NAC, creatinine clearance remained relatively stable (82 to 75 mL/min) and significantly declined to 48 mL/min postoperatively. This further emphasized that NAC is preferable to adjuvant therapy as patients may be rendered cisplatin-ineligible postoperatively [14].

The VESPER trial randomized 493 patients with MIBC to perioperative ddMVAC or gemcitabine/cisplatin (GC) in a 1:1 fashion. In the NAC cohort, patients received either six cycles of ddMVAC or four cycles of GC and were found to have similar pathological complete responses at the time of radical cystectomy (42% ddMVAC group and 36% in the GC cohort), though OS at 5 years was improved in those who received ddMVAC vs. GC (66% vs. 57%, HR 0.71). The 5-year cumulative incidence of death due to bladder cancer was also improved in the ddMVAC group (24% vs. 38%, HR 0.55). The data from the VESPER support the use of ddMVAC over GC in the neoadjuvant setting [15].

Currently, there is no role for neoadjuvant monotherapy immunotherapy in the treatment of UTUC, but this has been studied. The PURE-02 study treated patients with three cycles of pembrolizumab prior to RNU, and only one of the 10 patients had a radiographic CR [16].

Results from the iNDUCT-GETUG V08 phase II trial of non-metastatic, high-grade UTUC were recently published. Patients were treated with four cycles of either durvalumab + GC or durvalumab + gemcitabine/carboplatin (platinum choice dependent on glomerular filtration rate; cutoff was 60). In total, 50 patients were enrolled (31 randomized to the cisplatin arm and 19 to the carboplatin arm). The primary end point was the rate of pCR. Of the 45 patients who proceeded to RNU, 13% of the cisplatin arm achieved pCR compared to 5% of the carboplatin arm; 50% of the cisplatin arm were downstaged compared to 42% of the carboplatin arm. Though this was a negative study, the downstaging seen with NAC + immunotherapy is promising [17]. These findings led to the development of iNDUCT-3, a randomized phase III trial opening soon that is designed to formally evaluate NAC with immunotherapy in patients with high-risk localized UTUC.

## 4. Surgical Management

### 4.1. Endoscopic (Kidney-Sparing) Management (EM)

According to the AUA/SUO Guidelines, EM (retrograde ureteroscopy/pyeloscopy or antegrade ureteroscopy/pyeloscopy via percutaneous access with fulguration or ablation) is the recommended initial management option for those with low-risk, favorable disease. It can also be considered in patients with low-risk, unfavorable disease and in select patients with high-risk, favorable disease who have low-volume tumors, bilateral disease, or cannot undergo RNU (such as those who cannot due to comorbidities, poor renal function, or solitary kidney). In this setting, repeat endoscopic evaluation should be performed within three months, and ongoing surveillance with upper tract imaging, cystoscopy, and ureteroscopy should be performed.

A variety of endoscopic techniques can be used to ablate tumor tissue, including cautery/fulguration and laser ablation. It is important to note that anatomic limitations, the need for small ureteroscopes, and difficulty with irrigation and visualization can make endoscopic biopsy and ablation challenging. However, advances in technology have made this more feasible, and a variety of lasers have been studied in this space, including holmium/yttrium-aluminum-garnet (Ho:YAG), neodymium/YAG (Nd:YAG), and thulium/YAG (Tm:YAG). The Ho:YAG is an ideal laser for ureteral tumors, either alone or in combination with the others, given its depth of penetration of 0.2–0.5mm and less thermal injury to surrounding tissue compared to the other types. The different types of lasers also have variable coagulation abilities [18].

While EM offers the benefit of sparing the kidney, there are concerns regarding recurrence rates and the burden that ongoing surveillance places on patients and healthcare systems. A recent systematic review that included 2862 patients sought to characterize oncologic outcomes of patients with UTUC treated with EM. While there was heterogeneity in the studies included, the authors found that 1-, 2-, 5-, and 10-year OS rates were 96%, 87%, 80%, and 42%, respectively. The 1-, 2-, 5-, and 10-year CSS rates were 97%, 89%, 82%, and 69%, respectively. Recurrence-free survival (RFS) rates at 1, 2, and 5 years were 69%, 55%, and 45%, respectively. Intravesical recurrence-free survival (IVRFS) rates at 1, 2, and 5 years were 80%, 65%, and 64%, respectively. PFS rates at 2 and 5 years were 75% and 69%, respectively. Patients were also sub-stratified by tumor grade (i.e., low vs. high grade), and although there was much less data in the high-grade group, 2-year RFS was found to be 34% in this group compared to 52% in the low-grade group. In the low-grade group, 2-year IVRFS and PFS were 54% and 94%, respectively. The 2- and 5-year CSS and OS rates were 98% and 88% and 93% and 77%, respectively. Overall, the first two years after diagnosis appear to be critical in terms of risk of recurrence and progression, but EM can be safe in appropriately selected patients [19]. Other studies have shown similar excellent CSS rates in those with low-grade disease, regardless of whether they were treated with EM or extirpative surgery [20], and in those with larger tumors managed endoscopically [21].

If no bladder or ureteral perforation is noted, a single dose of intraurethral or intravesical chemotherapy may be administered to reduce the risk of recurrence. This is an optional practice that is extrapolated by the evidence for postoperative intravesical chemotherapy in UCB. The literature to support this practice is limited, and results are mixed, with some studies finding a trend towards reduced recurrences and others finding no difference [22,23].

EM is also not without burdens on the patient and the healthcare system. For example, one study found that those managed with EM had an average of three local recurrences and that the procedure burden and total cumulative time under anesthesia were significantly higher in this group compared to those managed with radical surgery. Overall, healthcare costs were higher in the EM group as well [24]. However, a recent systematic review found, in contrast, that RNU was overall more costly, taking into consideration costs of the procedure itself, readmissions, and long-term management of chronic kidney disease [25].

### 4.2. Radical Surgery

RNU with complete bladder cuff excision is considered the standard of care for patients with high-risk disease, low-risk but endoscopically unmanageable disease or risk group progression. The kidney, entire ureter, intramural tunnel, and ureteral orifice should be excised with watertight closure of the bladder. Formal bladder cuff excision has been shown in multiple studies to improve OS and CSS [26,27], but the evidence base is mixed and primarily based on retrospective series. Urine spillage should be avoided as much as possible. Lymph node dissection (LND) should be performed in those with high-risk disease and can be considered in those with low-risk disease. Whether LND confers oncologic benefit is somewhat uncertain. However, it allows for more accurate pathologic staging and therefore improved knowledge about prognosis and recurrence [28]. One study did find a potential benefit from LND regarding RFS in those with T2 and more advanced disease [29].

Distal ureterectomy and reimplantation can be performed for those with distal ureteral tumors, an ipsilateral normal kidney, and a normal bladder capacity. Segmental ureterectomy with ureteroureterostomy is not often performed but could also be considered in appropriate patients. A tension-free anastomosis is critical, and frozen sections of the ureteral margin should be sent to confirm complete resection.

A single dose of intravesical chemotherapy should be administered in the immediate postoperative setting to prevent recurrence in the bladder. Two prospective, randomized control trials showed that this practice reduces the risk of subsequent intravesical recurrence [30,31], as have retrospective studies [32].

Regarding operative approach for RNU, a randomized, prospective trial comparing laparoscopic and open RNU found that operative time and oncologic outcomes were similar between the two, but the laparoscopic group had lower mean blood loss and time to discharge. However, oncologic outcomes appeared to be improved in the open group for those with high-grade or more advanced tumors [33]. Either approach is generally acceptable and at the discretion of the surgeon, provided the oncologic and surgical principles are followed.

After surgical treatment, patients should be followed with regular upper tract imaging and cystoscopy as detailed in the AUA/SUO Guidelines.

### 4.3. Surgical Decision Making

While the AUA/SUO Guidelines offer several recommendations regarding appropriate management of the different UTUC disease states, there have been no randomized trials comparing kidney-sparing surgery (KSS) and RNU in patients with UTUC. In addition, several factors influence treatment decisions, such as the cancer characteristics, baseline renal function, and comorbidities, making each patient’s management unique.

A recent systematic review that included over 21,000 patients with localized UTUC sought to evaluate oncologic and renal function outcomes between those who received KSS (EM or segmental ureterectomy [SU]) and those who received RNU. Those who received KSS were less likely to have hydronephrosis, more likely to have low-grade tumors, and more likely to have a tumor stage of ≤pT1 compared to those who received RNU. The authors found no significant differences in 5-year OS, CSS, RFS, IVRFS, and MFS between the KSS and RNU groups. In addition, in the subgroup analysis comparing SU and RNU, there were no significant differences found in oncologic outcomes. However, in multivariate analysis, the EM group appeared to have worse OS as well as an increased risk of upper tract recurrence compared to the RNU group, which was statistically significant. Regarding renal function, while baseline (preoperative) renal function was similar between the two groups, the KSS group had significantly improved preservation of renal function compared to the RNU group [34].

Similarly, another systematic review found similar OS, CSS, and IVRFS rates between EM and RNU groups. However, they did note relatively high rates of upper tract recurrences (28–85%), more interventions with higher cumulative risk of complications, and longer cumulative time under anesthesia in those treated with EM compared to RNU. The overall combined recurrence rate for both the bladder and upper tract was also three times higher in those treated with EM compared to RNU [35]. A summary of surgical management of UTUC is shown in Table 2.

## 5. Adjuvant Intraluminal Therapy

In ongoing attempts to preserve patients’ renal function, intraluminal therapies have been developed and studied in patients with UTUC. A variety of agents have been assessed, primarily via retrospective studies, including chemotherapy and Bacillus Calmette–Guérin (BCG). Both retrograde (via reflux from a ureteral stent or direct injection via retrograde ureteroscopy/catheterization) and antegrade (via a nephrostomy tube) approaches have also been described. Techniques to overcome the issue of limited dwell time in the upper tract have also been evaluated, such as a slow-drip delivery method, gel formulations, and careful patient positioning.

A well-studied agent in this space is a reverse thermal polymer gel formulation of mitomycin (also known as UGN-101 or Jelmyto^®^). This agent polymerizes at body temperature and allows for longer contact time with the urothelium. The OLYMPUS trial, a multicenter, single-arm phase III trial, assessed the use of retrograde instillation of this agent as primary chemoablation in patients with measurable, low-grade UTUC and found 59% had a CR at the 3-month evaluation. However, adverse effects were not uncommon, including ureteral stenosis (44%), urinary tract infection (32%), hematuria (31%), and flank pain (30%) [36].

A retrospective analysis from multiple high-volume institutions assessed the efficacy and safety of this agent used as adjuvant therapy after visually complete endoscopic ablation or as primary chemoablation. The cohort had predominantly low-grade disease. Overall, 69% of patients in the adjuvant group (73% of those with initial low-grade disease and 50% of those with high-grade disease) had a CR at the first (~3-month) evaluation. Multifocality increased the risk of recurrence. This group also had low rates of progression (4%). In the primary chemoablation group, 37% had a CR at the first evaluation. Complete endoscopic ablation thus appeared to allow for both higher CR rates at the first evaluation as well as longer disease-free survival (DFS), as compared with chemoablation [37].

One study specifically assessed patients with carcinoma in situ (CIS) of the upper tract who were either treated with radical surgery or intraluminal BCG. The BCG was administered either through a nephrostomy tube, single-J ureteral stent, or double-J ureteral stent. The authors found no difference between the two groups in terms of RFS and CSS, suggesting that while radical surgery has been the gold standard, intraluminal BCG may be a reasonable alternative for patients with upper tract CIS [38]. Recent reviews have also supported this conclusion [39,40]. Intraluminal docetaxel has also been shown to be reasonably effective in those with BCG-refractory disease who decline or are unable to undergo radical surgery [41]. A meta-analysis found that in papillary disease (Ta/T1), there was no difference in several oncologic outcomes between BCG and mitomycin C, in contrast to the literature for bladder cancer, where BCG appears to be the most efficacious agent [42]. A summary of intraluminal therapies is shown in Table 3.

## 6. Adjuvant Systemic Therapy

The landmark trial proving the benefit of adjuvant chemotherapy in UTUC is POUT: a phase II, randomized, open-label trial of 261 patients with non-metastatic UTUC who were randomized to either platinum-based chemotherapy or surveillance post-RNU. Amongst those randomized to adjuvant therapy, patients received gemcitabine plus either cisplatin or carboplatin (based on glomerular filtration rate). Improvement in DFS at 2 years was seen in the adjuvant chemotherapy arm (70% vs. 51%). Non-cisplatin-based regimens did not significantly impact mortality outcomes. Final data were published in 2024: 5-year OS was 66% in the adjuvant therapy arm and 57% in the surveillance arm, univariable HR, 0.68 (95% CI, 0.46–1.00, *p* = 0.049). The difference in restricted mean survival time between the two groups was 11 months (95% CI, 1–21). This study solidified the recommendation for adjuvant platinum-based chemotherapy in localized UTUC, with the cisplatin-based regimen preferred if eligible [43].

CheckMate 274 was a phase III, multicenter, double-blinded, randomized, controlled trial of 709 urothelial carcinoma patients, 353 who received one year of adjuvant nivolumab and 356 who received a placebo. Receipt of NAC was allowed. 149 of 709 total patients had UTUC, and subgroup analysis showed no benefit with adjuvant nivolumab in this group. Though this study was not powered to specifically evaluate UTUC, these findings led to a general conclusion that adjuvant nivolumab is not recommended in UTUC [44].

The AMBASSADOR trial, a phase III, double-blind, placebo-controlled clinical trial, randomized 702 patients with high-risk muscle-invasive urothelial carcinoma (154 of whom had UTUC) to one year of adjuvant pembrolizumab or observation. Subgroup analysis of the UTUC cohort did not show a benefit of adjuvant immunotherapy [45]. Adjuvant atezolizumab was also explored in the IMvigor010 study. A total of 809 patients with localized urothelial carcinoma (54 with UTUC) were enrolled and randomized to receive adjuvant atezolizumab or observation. Similar to the AMBASSADOR study, no DFS benefit was observed in the UTUC group [46]. A summary of adjuvant systemic therapy is shown in Table 4.

## 7. Systemic Therapy in Metastatic Disease

The treatment of advanced and metastatic UTUC mirrors that of metastatic UCB. Given the relative rarity of UTUC, these tumors are often included alongside UCB in clinical trials. Some UTUC patients have functionally solitary kidneys or renal dysfunction secondary to the primary tumor, which may put them at a higher risk of chemotherapy-induced nephrotoxicity. This and additional factors, such as performance status, medical comorbidities, and organ function, must be taken into account in the choice of systemic therapy.

The combination of enfortumab vedotin (EV) and pembrolizumab has largely replaced platinum-based chemotherapy in the front-line treatment of advanced and metastatic urothelial carcinoma. The EV-302 study randomized patients with previously untreated locally advanced or metastatic urothelial carcinoma to receive either EV-pembrolizumab or platinum-based chemotherapy with gemcitabine and either cisplatin or carboplatin. The OR rate was 67.7% [95% CI, 63.1–72.1] with EV-pembrolizumab and 44.4% [95% CI, 39.7–49.2; *p* < 0.001] with chemotherapy, including CRs in 29.1% and 12.5% of patients, respectively. Median OS was 31.5 mos [95% CI, 25.4–not estimable] with EV-pembrolizumab and 16.1 mos [95% CI, 13.9–18.3] with chemotherapy (HR, 0.47 [95% CI, 0.38–0.58]; *p* < 0.001). Median PFS was 12.5 mos [95% CI, 10.4–16.6] vs. 6.3 mos [95% CI, 6.2–6.5] (HR, 0.45 [95% CI, 0.38–0.54]; *p* < 0.001). Additionally, 30.5% of patients in the enfortu EV-pembrolizumab arm and 23.4% in the chemotherapy arm had UTUC. OS and PFS improved with EV-pembrolizumab across all subgroups, including patients with UTUC and in cisplatin-eligible and cisplatin-ineligible patients. Among patients with UTUC, median OS was not estimable with EV-pembrolizumab and 18.4 mos with chemotherapy; median PFS was 12.7 mos vs. 6.2 mos. For cisplatin-eligible patients, median OS was 31.5 mos vs.18.4 mos; median PFS was 14.6 vs. 6.5 mos. In cisplatin-ineligible patients, median OS was not estimable vs.12.7 mos, and median PFS was 10.6 mos vs. 6.1 mos [47].

Cohort K in the phase Ib/II EV-103 study looked at EV vs. EV-pembrolizumab amongst cisplatin-ineligible patients with previously untreated advanced/metastatic urothelial carcinoma. There was a significant improvement in OR rates amongst patients receiving the doublet therapy compared to those receiving EV alone [64.5% (95% CI, 52.7–75.1) and 45.2% (95% CI, 33.5–57.3)] [48].

For patients who decline or are ineligible to receive EV due to significant motor or sensory neuropathy, poorly controlled diabetes, or a significant dermatologic condition, chemoimmunotherapy can be considered in the front-line. The CheckMate-901 study evaluated the use of gemcitabine, cisplatin, and nivolumab compared to GC in patients with previously untreated unresectable or metastatic urothelial carcinoma. Median OS was 21.7 mos [95% CI, 18.6–21.7] with nivolumab–chemotherapy and 18.9 mos [95% CI, 14.7–22.4] in the GC group. Median PFS was 7.9 mos [95% CI, 0.59–0.88] in comparison to 7.6 mos [95% CI, 6.1–7.8] (HR, 0.72 [95% CI, 0.59–0.88]; *p* = 0.001). Hazard ratios favored the nivolumab arm across most subgroups, including patients with PD-L1 expression ≥1%, where the HR for OS was 0.75 [95% CI, 0.53–1.06] and for PFS was 0.60 [95% CI, 0.41–0.81], and those with ECOG performance status 0, where the HR for OS was 0.70 [95% CI, 0.51–0.95]. The OS benefit in patients without liver metastases, where the HR was 0.77 [95% CI, 0.61–0.98], may be relevant to renal pelvis urothelial carcinoma, as these tumors typically spread to lymph nodes rather than the liver. UTUC was included in the study, comprising 10.9% of the nivolumab arm and 14.5% of the standard-of-care arm [49].

Platinum-based chemotherapy remains a treatment option for patients who are not candidates for or who have progressed while on EV and pembrolizumab. These regimens yield response rates of close to 50%, median OS of 13–15 months, and median PFS of 7 months in the front line [50]. MVAC demonstrated a significant improvement in response rate compared with single-agent cisplatin amongst patients with metastatic urothelial carcinoma (39% vs. 12%, *p* < 0.0001) [51]. There was also an improved OS (12.5 vs. 8.2 mos) and PFS (10.0 vs. 4.3 mos). The MVAC regimen was, however, more toxic, with a higher rate of myelosuppression, cytopenias, and mucositis. Dose-dense MVAC (ddMVAC) led to improved response rates (62% [95% CI, 54–70%] vs. 50% [95% CI, 42–59%]) and PFS (9.1 vs. 8.2 months [*p* = 0.037]), compared with traditional MVAC [52,53]. The dose-dense arm also had a better toxicity profile. There was no statistically significant difference in OS, however. The GC combination is another treatment option for cisplatin-eligible patients who are not candidates for EV and pembrolizumab. A phase III study comparing GC with MVAC in patients with advanced or metastatic UCB found similar rates of OS, time to progression, and response rates [50,54]. However, toxicity was higher with MVAC.

For cisplatin-ineligible patients who are not candidates for EV and pembrolizumab, carboplatin-based treatment is an option. A study comparing treatment with gemcitabine and carboplatin with methotrexate/carboplatin/vinblastine amongst patients with glomerular filtration rates of 30–60 mL/min found similar rates of OS and PFS between the arms, though with a non-statistically significant improved response rate (41.2% vs. 30.3%, *p* = 0.08) [55].

Maintenance avelumab is an option for patients with advanced or metastatic urothelial carcinoma who did not progress on first-line platinum-based chemotherapy. The JAVELIN Bladder 100 study randomized metastatic urothelial carcinoma patients who had at least stable disease after four to six cycles of chemotherapy with gemcitabine and cisplatin or carboplatin to receive either maintenance avelumab or best supportive care. The study included patients with both upper and lower urinary tract disease, with 26% having UTUC. The median OS was 21.4 mos [95% CI, 18.9–26.1] in the avelumab group and 14.3 mos in the best support care group [95% CI, 12.9–17.9]. Median PFS was also improved with avelumab, 3.7 mos [95% CI, 3.5–5.5] vs. 2.0 mos [95% CI, 1.9–2.7] [56].

Pembrolizumab is approved for use in the front line for patients with advanced or metastatic urothelial carcinoma who are not candidates for any platinum-based chemotherapy based on the results of the KEYNOTE-052 trial, which found OR rates of 28.6%, with a CR rate of 8.9% [57,58].

Fibroblast growth factor receptor (FGFR) alterations are found in around 20% of metastatic urothelial carcinomas, though they are more frequent amongst UTUC, with an estimated frequency of 30–36% [59,60]. Erdafinitinib was granted FDA-approval in 2019 for patients with advanced or metastatic urothelial carcinoma with FGFR 3 or 2 alterations who had progressed post-platinum chemotherapy based on the results of the phase II BLC2001 study, which found an OR rate of 40% (95% CI, 31 to 50), PFS of 5.5 mos (95% CI, 4.2–6.0), and an OS of 13.8 mos (95% CI, 9.8 to not reached) [61]. The follow-up phase III THOR trial compared the use of erdafitinib to chemotherapy (docetaxel or vinflunine) amongst patients with advanced or metastatic urothelial carcinoma with qualifying FGFR2/3 alterations after one or two prior lines of systemic therapy, which included anti-PD-L1 or PD-1 agents. In total, 30% of patients on the erdafitinib arm and 36.9% on the chemotherapy arm had UTUC. Median OS was 12.1 mos [95% CI, 10.3–16.4] in the erdafitinib arm and 7.8 mos [95% CI, 6.5–11.1] in the chemotherapy arm. Median PFS was 5.6 mos [95% CI, 4.4–5.7] with erdafitinib and 2.7 mos [95% CI, 1.8–3.7] with chemotherapy, with an OR rate of 45.6% vs. 11.5% between the erdafitinib and standard-of-care arms [95% CI, 2.37–6.57; *p* < 0.001] [62].

Fam-trastuzumab deruxtecan (Enhertu) is a treatment option for patients with HER2 overexpression (IHC3+) who have progressed on prior systemic therapy and for whom there is no satisfactory disease-specific alternative treatment. The DESTINY-PanTumor02 study was a phase II trial that demonstrated an OR rate of 37.1% [95% CI, 31.3–43.2] for the overall population, with a response rate of 39% [95% CI, 24.2 to 55.5] for the cohort with UCB. For UCB patients with HER2 IHC3+, the response rate was 56.3%. Although this study included only patients with metastatic UCB, the drug has tumor-agnostic approval and can be used to treat UTUC [63].

**Figure 1:**
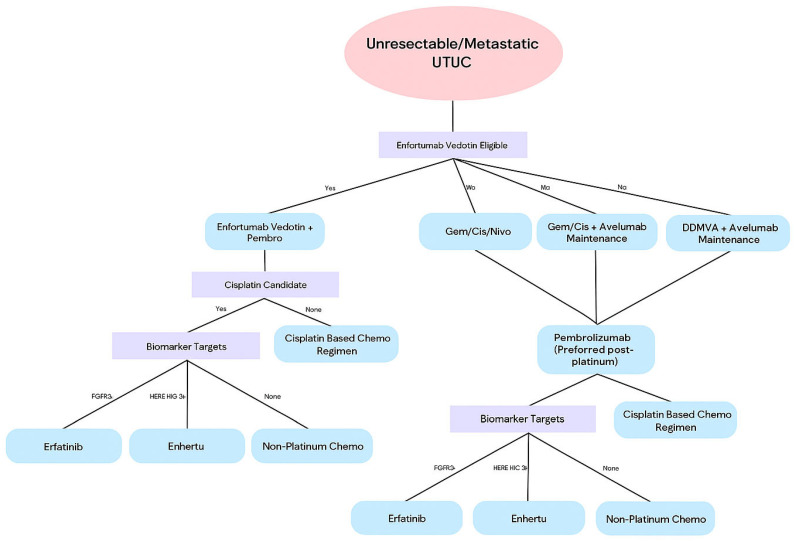
Flowchart for systemic therapy in unresectable or metastatic upper tract urothelial carcinoma (UTUC).

A flowchart summary of systemic therapy in unresectable or metastatic UTUC is shown in Figure 1 and Table 5.

## 8. Conclusions and Future Directions

UTUC is a rare but clinically aggressive malignancy associated with poor outcomes and limited treatment options. Despite growing interest, current management strategies remain largely extrapolated from UCB studies, failing to address the unique biology and clinical challenges of UTUC.

Accurate preoperative staging and risk stratification are crucial for guiding treatment decisions, yet standard diagnostic approaches have technical limitations and are further hindered by intratumoral grade heterogeneity. A pooled meta-analysis of 23 studies reported under-grading and under-staging rates of 32% and 46%, respectively [64]. These diagnostic shortcomings are particularly problematic as emerging evidence supports the use of NAC prior to RNU, a procedure that often compromises renal function and limits eligibility for postoperative cisplatin-based therapy. Recent advancements in diagnostics and treatment offer encouraging progress toward more precise, individualized care. A prospective study evaluating plasma-derived circulating tumor DNA (ctDNA) profiling in high-risk UTUC demonstrated 71% sensitivity for detecting muscle-invasive or node-positive disease, with ctDNA positivity also correlating with significantly worse one-year PFS and CSS [65]. These findings suggest that ctDNA may serve as a valuable noninvasive biomarker for preoperative risk assessment.

The use of mRNA expression will likely be a cornerstone in terms of risk stratification and predictive responses to variable treatment. Robertson et al. studied mRNA expression clustering and were able to classify urothelial carcinoma samples into subtypes, including luminal papillary, luminal infiltrated, luminal, or basal/squamous, then used the classification to choose appropriate treatment. It is possible that, as therapeutics become more personalized, this method of mRNA will guide the future of systemic treatment of urothelial carcinoma [66]. Chu et al. had a similar approach of using mRNA expression along with mutations and epigenomic DNA methylation information to create subtypes of MIBC through consensus machine learning-driven signatures to predict prognosis and responsiveness to immunotherapy, again with the end goal of being a precision-based classification to guide personalized treatment [67].

A growing body of evidence supports the integration of biomarker-driven, platinum-free regimens in the perioperative management of UTUC. A recent case report described a pathologic CR in a cisplatin-ineligible patient treated with neoadjuvant pembrolizumab and EV, with the addition of olaparib guided by somatic mutation profiling—highlighting the potential of precision oncology in this setting [68]. Similarly, a recent real-world retrospective study demonstrated that adjuvant therapy with disitamab vedotin and toripalimab in patients with HER2-overexpressing UTUC resulted in a 12-month DFS rate of 91.7%, compared to 62.5% in those without adjuvant treatment. This therapy was well tolerated with no grade ≥3 adverse events [69]. These findings reflect a paradigm shift toward personalized, molecularly informed treatment strategies.

To further explore the role of novel immunotherapy-based regimens, several prospective trials are underway. The NEPTUNE trial is an approved phase II study evaluating neoadjuvant EV plus pembrolizumab in patients with UTUC [70], while a separate phase II trial is actively investigating this same combination with pembrolizumab use before and after RNU in patients with high-risk UTUC [71]. The EV-103 phase Ib/II clinic trial included cisplatin-ineligible patients with cT2-T4aN0M0 disease who received neoadjuvant EV followed by radical cystectomy. The pathologic downstaging rate was 50%, the pCR rate was 36.4%, and the event-free survival (EFS) rate was 62% at 2 years [72]. The KEYNOTE-905/EV-303 phase III trial is assessing perioperative pembrolizumab alone or in combination with EV in T2-T4aN0M0 or T1-T4aN1M0 UCB patients who are ineligible for or decline cisplatin-based therapy [73]. The KEYNOTE-B15/EV-304 phase III trial is assessing the combination of EV-pembrolizumab in cisplatin-eligible patients with T2-T4aN0M0 or T1-T4aN1M0 UCB. The primary endpoints for these trials are pCR and EFS [74]. These results will likely guide future therapy in UTUC as well.

In summary, UTUC remains a challenging malignancy with distinct diagnostic and therapeutic needs that are not adequately addressed by current UCB-based treatment paradigms. Advances in noninvasive diagnostics, such as ctDNA profiling, and promising early results from neoadjuvant and adjuvant immunotherapy trials highlight a shift towards therapeutic regimens more specific to UTUC.

## Figures and Tables

**Table 1:** Risk stratification in upper tract urothelial carcinoma (adapted from AUA guidelines).

**Feature**	Low Risk		High Risk	
**Histologic Grade**	Low grade		High grade	
**Sub-stratification**	Favorable	Unfavorable	Favorable	Unfavorable
**Cytology**	Negative	No HGUC	Any	HGUC
**Imaging**	No invasion, obstruction, or LAD	No invasion or LAD; Yes, obstruction	No invasion, obstruction, or LAD	Yes, invasion, obstruction, suspicious LAD
**Focality**	Unifocal	Multifocal	Unifocal	Multifocal
**Appearance**	Papillary	Papillary	Papillary	Sessile or flat
**Lower Tract Involvement**	No	Yes	No	Yes

HGUC = high-grade urothelial carcinoma; LAD = lymphadenopathy.

**Table 2:** Surgical management of UTUC.

	**Indications**	**Surgical Approach**	**Intravesical Chemotherapy**	**Oncologic Outcomes**	**Surveillance**
**Endoscopic Management (EM)**	- low-risk disease - select high-risk, favorable patients	- retrograde ureteroscopy - antegrade ureteroscopy with percutaneous renal access - ablation with cautery or laser	- recommended in perioperative setting	- 5-year OS: 8% - 5-year CSS: 82% - 2-year RFS: 55% - 2-year PFS: 75%	- note: high risk of recurrence requiring several procedures under anesthesia - repeat endoscopic evaluation in 3 months - cystoscopy - upper tract imaging
**Radical Surgery (RNU or SU)**	- high-risk disease - endoscopically unmanageable disease - risk group progression - SU: distal tumor with ipsilateral normal kidney, normal bladder capacity	- open or minimally invasive - formal bladder cuff excision is critical - LND should be performed	- recommended in perioperative setting	- similar to EM - evidence shows improved OS and decreased recurrences with RNU compared to EM - similar outcomes between RNU and SU	- cystoscopy - upper tract imaging

EM = endoscopic management; OS = overall survival; CSS = cancer-specific survival; RFS = recurrence-free survival; PFS = progression-free survival; RNU = radical nephroureterectomy; SU = segmental ureterectomy; LND = lymph node dissection.

**Table 3:** Adjuvant intraluminal therapy.

Instillation Approach	Agents Studied	Evidence
- retrograde: via reflux from a ureteral stent or direct injection via retrograde ureteroscopy/catheterization - antegrade: via nephrostomy tube - to increase dwell time: slow-drip delivery, gel formulations, patient positioning - chemoablation vs. adjuvant after complete endoscopic ablation	- BCG - mitomycin (Jelmyto^®^) - docetaxel	- OLYMPUS trial: Jelmyto^®^ as chemoablation; 59% CR at 3 months; high rates of AE - adjuvant use 69% CR at 3 months, 4% progression - similar outcomes between RNU and intraluminal BCG in CIS - similar outcomes between BCG and mitomycin in papillary disease

BCG = Bacillus Calmette–Guérin; CR = complete response; AE = adverse event.

**Table 4:** Summary of adjuvant systemic therapy options vs. observation in UTUC.

Study	Adjuvant Therapy	Population	Disease Free Survival
POUT	Platinum-Based Chemo	261 patients w/non-metastatic UTUC	DFS at 5 years: 66% vs. 57%
CheckMate 274	Nivolumab	709 patients with MIBC, including 149 with UTUC	No DFS benefit in UTUC subgroup
AMBASSADOR	Pembrolizumab	702 patients with MIBC, including 154 with UTUC	No DFS benefit in UTUC subgroup
IMvigor010	Atezolizumab	809 patients with MIBC, including 54 with UTUC	No DFS benefit in UTUC subgroup

**Table 5:** Summary of systemic therapy options in urothelial carcinoma.

Study	Systemic Therapy	Median OS	Key Outcomes
EV-302	Enfortumab Vedotin vs. Platinum-Based Chemo	31.5 months vs. 16.1 months	Median OS: not estimable vs. 18.4 months in UTUC subgroup
CheckMate 901	Gem/Cis + Nivolumab vs. Gem/Cis	21.7 months vs. 18.9 months	UTUC comprised 10.9% of nivolumab arm and 14.5% of the chemo arm
von der Maase H et al.	Gem/Cis vs. ddMVAC	13.8 months vs. 14.8 months	Gem/Cis has comparable OS to ddMVAC w/ better safety profile
JAVELIN Bladder 100	Maintenance Avelumab post Platinum Chemo vs. Observation	21.4 months vs. 14.3 months	UTUC comprised 26% of study population
KEYNOTE 052	Pembrolizumab	11.3 months	Approved for patients ineligible for any platinum-containing therapy
THOR	Erdafitinib vs. Chemo	12.1 months vs. 7.8 months	Approved for patients with FGFR2/3 mutations
DESTINY-PanTumor02	Trastuzumab deruxtecan (T-DXd)	12.8 months	Approved for HER2-positive IHC3+
